# Supplementary material for: Subcutaneous mycoses: Endemic but neglected among the Neglected Tropical Diseases in Ethiopia
Source: PLoS Negl Trop Dis. 2023 Sep 27;17(9):e0011363. doi: 10.1371/journal.pntd.0011363 (PMC10561858; doi:10.1371/journal.pntd.0011363)
Supplement: Attachment — Submitted filename: Point by point response for reviewer Comments_2872023.docx [file pntd.0011363.s003.docx]

Dear editors and reviewers

Thank you for the constructive inputs.

We would like to draw your attention to the fact that since submission of the paper, we have received additional data from a regional hospital in the North of the country.

During our initial data collection, the region (Tigray) was inaccessible due to the two-year-long conflict in Northern Ethiopia. However, following the peace agreement, the region and the referral hospital have become accessible. We have included this data to ensure comprehensive representation of the major regions in the country and its significance for national planning. The overall findings of the paper remain unaltered, but we now have a more complete picture of the burden in the country. We hope you agree with this approach.

Regarding the reviewer's comment, we have thoroughly reviewed the manuscript (attached with track changes), and below you will find our point-by-point response:

**Introduction**

- Expand on the context of the study, public health implications of subcutaneous mycoses?

A: Thank you and we have added a paragraph on the impact

**Study Design**

- Description very limited. Expand on the mixed methods approach- rapid assessment used in combination with quantitative outcomes

A: We have used only a cross sectional quantitative method and no qualitative.

**Selection of Hospitals**

- Characteristics in the study area that predisposes patients to subcutaneous mycoses should have been elucidated.

We have documented Ethiopia is located in the mycetoma belt and bordering Sudan (the largest mycetoma reporting country in the world) but specific information for the different regions in the country does not exist. The Hospitals are selected based on convenience and regional representations as part of the rapid assessment and expert consensus to assist national mapping of the disease.

- “retrospective data collection, we included the main referral hospital or dermatology center in the regions or administrative town,”. Are all the hospitals in Table 1 tertiary hospitals? Indicate tier level

No all the hospitals are not tertiary hospitals and the tier of each hospital is included in the names of the hospitals plus we have added the catchment populations of each hospitals. With a prior knowledge of the centers which are serving as a dermatology referral to the catchment population, the Hospitals are selected based on convenience and regional representations

**Data Collection**

- The data collection method should be described more in detail to provide a general idea of proportion. 85 cases of subcutaneous mycoses were found from what total number of documentation from July 2018 to September 2022? Or was data collection specifically from clinical notes or lab results that had a diagnosis of a subcutaneous mycosis? What is the population that these 12 hospitals serve?

Correct, forthis rapid assessment, we scanned diagnoses of subcutaneous mycosis/mycetoma/chromoblastomycosis and sporotrichosis from the outpatient registrations, which was validatedby review of the patient clinical records. Hence, we do not have information on the proportion of subcutaneous mycoses amongst all patient consultations. We have added the numbers on catchment population served in the Table.

- No statements provided on deidentification and confidentiality of data.

Thank you we have added statement for that

**Ethics statement**

- Apart from the support letter from the Federal MOH of Ethiopia was an ethical approval of the study not provided from any authority?

As the project was an integral part of the Ministry of Health for health system planning a formal ethical clearance was not needed

**Results**

- Was a combination of clinical presentation, imaging and microscopic laboratory investigations used to confirm diagnosis or was this provided independently of each other? I think this should be clarified.

Most of the diagnosis is clinical (since there is no facility in the country to do species identification) which we have explained as limitation ….the laboratory investigations and imaging are just supportive and the data from Tigray we have received only the diagnosis with no information on the investigation (this is also mentioned in the discussion)

- A definition of methodology of diagnosis would be important. To be on the same page with the readers.

Thanks and we have added the information on methodology: The diagnosis of subcutaneous mycosis was considered if the register contains; mycetoma, chromoblastomycosis, sportotrichosis and deep fungal infection. The laboratory and imaging information’s were collected (if they are found) as supportive evidences.

A number of grammatical errors noted, to be reviewed.

We have fully reviewed the paper for language and have made editorial changes throughout.

This is a great and informative study. It covers an aspect that is so much neglected- burden of subcutaneous mycoses in SSA. This can only be determined though through accurate identification of the causative agents therefore an urgent need for cost-effective, accurate and reliable diagnostic tests which should be stressed upon in the conclusions.
