# Supplementary material for: Subcutaneous mycoses: Endemic but neglected among the Neglected Tropical Diseases in Ethiopia
Source: PLoS Negl Trop Dis. 2023 Sep 27;17(9):e0011363. doi: 10.1371/journal.pntd.0011363 (PMC10561858; doi:10.1371/journal.pntd.0011363)
Supplement: Attachment — Submitted filename: Reviewer Comments.docx [file pntd.0011363.s002.docx]

**Introduction**

- Expand on the context of the study, public health implications of subcutaneous mycoses?

**Study Design**

- Description very limited. Expand on the mixed methods approach- rapid assessment used in combination with quantitative outcomes

**Selection of Hospitals**

- Characteristics in the study area that predisposes patients to subcutaneous mycoses should have been elucidated.
- “retrospective data collection, we included the main referral hospital or dermatology center in the regions or administrative town,”. Are all the hospitals in Table 1 tertiary hospitals? Indicate tier level

**Data Collection**

- The data collection method should be described more in detail to provide a general idea of proportion. 85 cases of subcutaneous mycoses were found from what total number of documentation from July 2018 to September 2022? Or was data collection specifically from clinical notes or lab results that had a diagnosis of a subcutaneous mycosis? What is the population that these 12 hospitals serve?
- No statements provided on deidentification and confidentiality of data.

**Ethics statement**

- Apart from the support letter from the Federal MOH of Ethiopia was an ethical approval of the study not provided from any authority?

**Results**

- Was a combination of clinical presentation, imaging and microscopic laboratory investigations used to confirm diagnosis or was this provided independently of each other? I think this should be clarified.
- A definition of methodology of diagnosis would be important. To be on the same page with the readers.

A number of grammatical errors noted, to be reviewed.

This is a great and informative study. It covers an aspect that is so much neglected- burden of subcutaneous mycoses in SSA. This can only be determined though through accurate identification of the causative agents therefore an urgent need for cost-effective, accurate and reliable diagnostic tests which should be stressed upon in the conclusions.
